# Royal College of Surgeons of England

**Published:** 1852-11

**Authors:** 


ROYAL COLLEGE OF SURGEONS OF ENGLAND.

*Lincoln's Inn Fields.*

*President.*—Cæsar H. Hawkins.

*Vice-Presidents.*—James Luke, and Robert Keate.

*The Council.*—The President and Vice-Presidents, G. J. Guthrie, T. Copeland, W. Lawrence, Sir B. C. Brodie, Bart., B. Travers, J. Swan, J. H. Green, E. Stanley, J. M. Arnott, J. F. South, F. C. Skey, B. B. Cooper, J. Hodgson, T. Wormald, G. Pilcher, J. Bishop, G. W. Mackmurdo, F. Kiernan, W. Coulson, G. Gulliver, and R. Partridge.

*Court of Examiners.*—The President and Vice-Presidents, G. J. Guthrie, W. Lawrence, B. Travers, E. Stanley, J. H. Green, J. M. Arnott, John F. South.

*Examiners for the Fellowship in Classics, Mathematics, and French.* G. Smith, G. G. Stokes, and I. Brasseur.

*Professor of Anatomy and Physiology.*—F. C. Skey.

*Hunterian Professor,*<br>*Conservator of Museum.* } R. Owen.

*Conservator.*—J. T. Quekett.

*Librarian.*—T. M. Stone.

*Secretary.*—E. Belfour.

*Clerk.*—H. P. Gregg.

*Mace-bearer.*—W. Stone.

*Regulations of the Council respecting the Professional Education of Candidates for the Diploma of Member of the College.*

I. Candidates will be required to produce the following Certificates, viz.—

1. Of being twenty-one years of age.

2. Of having been engaged during four years in the acquirement of professional knowledge.

3. Of having studied practical pharmacy during six months.

4. Of having attended at a recognised hospital or hospitals in the United Kingdom the practice of physic during one winter* and one summer† session.

5. Of having attended, during three winter and two summer sessions, the practice of surgery at a recognised hospital or hospitals in the United Kingdom.

6. Of having studied anatomy and physiology, by attendance on lectures and demonstrations, and by dissections, during three winter sessions.

7. Of having attended, during two winter sessions, lectures on the principles and practice of surgery.

8. Of having attended, during one summer session, lectures on materia medica, and lectures on midwifery; practical midwifery to be attended at any time after the conclusion of the session.

9. And of having attended one course of lectures on the practice of physic, and one course on chemistry.

*** The course of study hereby prescribed is required to be observed by candidates who shall have pursued their studies in hospitals and schools in England. Those candidates who shall have studied in Scotland are required to bring certificates of having attended lectures on the

* The winter session comprises a period of six months, and, in England, commences on the 1st of October, and terminates on the 31st of March.

† The summer session comprises a period of three months, and, in England, commences on the 1st of May, and terminates on the 31st of July.

No provincial hospital will be recognized by this College which contains less than 100 patients; and no metropolitan hospital which contains less than 150 patients.

institutes of medicine during one winter session, and on anatomy during two other winter sessions, and on demonstrations and dissections during three winter sessions, (the foregoing regulations being in all other respects observed.) Candidates who shall have attended lectures on materia medica in the University of Dublin will be allowed to bring certificates of such attendance during the winter session.

II. Members or licentiates of any legally constituted College of Surgeons in the United Kingdom, and graduates in surgery of any university requiring residence to obtain degrees, will be admitted for examination on producing their diploma, licence, or degree, together with proof of being twenty-one years of age, and of having been occupied at least four years in the acquirement of professional knowledge.

III. Graduates in medicine of any legally constituted college or university requiring residence to obtain degrees, will be admitted for examination on adducing, together with their diploma or degree, proof of having completed the anatomical and surgical education required by the foregoing regulations, either at the school and hospital of the university where they shall have graduated, or at one or more of the recognised schools and hospitals in the United Kingdom.

IV. Candidates who shall have attended at recognised colonial hospitals and schools,* the medical and surgical practice and the several courses of lectures, with the demonstrations and dissections, required by the foregoing regulations, will be admitted for examination upon producing certificates of such attendance, together with certificates of having attended in London, during one winter session, the surgical practice of a recognised hospital, and lectures on anatomy, physiology, and surgery, with demonstrations and dissections.

V. Certificates will not be recognized from any hospital unless the surgeons thereto be members of one of the legally constituted Colleges of Surgeons in the United Kingdom; nor from any school of anatomy and physiology or midwifery, unless the teachers in such school be members of some legally constituted College of Physicians or Surgeons in the United Kingdom; nor from any school of surgery, unless the teachers in such school be members of one of the legally constituted Colleges of Surgeons in the United Kingdom.

VI. Certificates will not be received on more than one branch of science from one and the same lecturer: but anatomy and physiology—demonstrations and dissections—will be respectively considered as one branch of science; and in those schools in Scotland or Ireland in which such division of those subjects is sanctioned by the College of Surgeons in each kingdom, the institutes of medicine,—anatomy, demonstrations, and dissections,—may be separately certified.

VII. Certificates will not be received from candidates who have studied in London, unless they shall have registered their tickets at the

*The recognition of colonial hospitals and schools is governed by the same regulations, with respect to number of patients, to courses of lectures, and to physicians, surgeons, and lecturers, as apply to the recognition of provincial hospitals and schools in England.

College, as required by the regulations, during the last ten days of January, March, and October in each year; nor from candidates who have studied elsewhere, unless their names shall duly appear in the registers transmitted during such studies from their respective schools.

By order of the Council,

*July 1st,* 1852. EDMUND BELFOUR, Secretary.

N. B. In the certificates of attendance on hospital practice and on lectures, it is required that the dates of commencement and termination be clearly expressed; and no interlineation, erasure, or alteration will be allowed.

Blank forms of the required certificates may be obtained on application to the secretary, to whom they must be delivered, properly filled up, ten days before the candidate can be admitted to examination; and all such certificates are retained at the College.

